# Rural to urban migration and changes in cardiovascular risk factors in Tanzania: a prospective cohort study

**DOI:** 10.1186/1471-2458-10-272

**Published:** 2010-05-24

**Authors:** Nigel Unwin, Peter James, Dorothy McLarty, Harun Machybia, Peter Nkulila, Bushiri Tamin, Mkay Nguluma, Richard McNally

**Affiliations:** 1 Institute of Health and Society, Newcastle University, Newcastle, NE2 4AX, UK; 2 Regional Medical Officer, PO Box 259, Mbeya, Tanzania; 3 Morogoro Regional Hospital, PO Box 110, Morogoro, Tanzania; 4 Muhimbili University of Health and Allied Sciences, PO Box 65001, Dar es Salaam, Tanzania

## Abstract

**Background:**

High levels of rural to urban migration are a feature of most African countries. Our aim was to investigate changes, and their determinants, in cardiovascular risk factors on rural to urban migration in Tanzania.

**Methods:**

Men and women (15 to 59 years) intending to migrate from Morogoro rural region to Dar es Salaam for at least 6 months were identified. Measurements were made at least one week but no more than one month prior to migration, and 1 to 3 monthly after migration. Outcome measures included body mass index, blood pressure, fasting lipids, and self reported physical activity and diet.

**Results:**

One hundred and three men, 106 women, mean age 29 years, were recruited and 132 (63.2%) followed to 12 months. All the figures presented here refer to the difference between baseline and 12 months in these 132 individuals. Vigorous physical activity declined (79.4% to 26.5% in men, 37.8% to 15.6% in women, p < 0.001), and weight increased (2.30 kg men, 2.35 kg women, p < 0.001). Intake of red meat increased, but so did the intake of fresh fruit and vegetables. HDL cholesterol increased in men and women (0.24, 0.25 mmoll^-1 ^respectively, p < 0.001); and in men, not women, total cholesterol increased (0.42 mmoll^-1^, p = 0.01), and triglycerides fell (0.31 mmoll^-1^, p = 0.034). Blood pressure appeared to fall in both men and women. For example, in men systolic blood pressure fell by 5.4 mmHg, p = 0.007, and in women by 8.6 mmHg, p = 0.001.

**Conclusion:**

The lower level of physical activity and increasing weight will increase the risk of diabetes and cardiovascular disease. However, changes in diet were mixed, and may have contributed to mixed changes in lipid profiles and a lack of rise in blood pressure. A better understanding of the changes occurring on rural to urban migration is needed to guide preventive measures.

## Background

Since 2008, and for the first time in human history, over 50% of the world's population lives in urban areas[1]. In sub Saharan Africa it is estimated that 35% of the population lived in urban areas in 2005 and that by 2050 it will have risen to 61%[2]. In Tanzania, the location of the study described here, the urban population is increasing at a rate of 4.2% per year, compared to 1.9% for the rural population[2]. A little under 30% of Tanzania's 9.3 million urban dwellers live in the city of Dar es Salaam, its main commercial centre[2].

In low and middle income countries, urban compared to rural living is strongly associated with a higher prevalence of certain chronic, non-communicable, conditions, including hypertension, glucose intolerance, obesity and dyslipidaemia[3,4]. Within Tanzania, we have previously described higher prevalences of diabetes, obesity[5], and hypertension [6], and higher levels of mortality from chronic non-communicable diseases [7], particularly from stroke[8] in Dar es Salaam compared to rural areas. The main determinants thought to underlie these rural to urban differences are changes in physical activity and diet, often referred to as the "nutrition transition"[9].

Despite these well known rural to urban differences there are scant data on the speed, size and determinants of changes that occur on rural to urban migration in regions such as sub Saharan Africa. Our overall goal in establishing the Urbanization and Metabolic Outcomes Study in Tanzania (UMOST) was to determine if changes in blood pressure and lipids occurred within the first twelve months of rural to urban migration, and if so what determinants underlay those changes. The first phase of this study, on which this report is based, was designed to estimate the direction, size and variance of any changes, and help to generate hypotheses on the determinants of those changes. In a preliminary short report from this study, using data from two time points, baseline and 6 months, we described changes in a number of biological variables over the first six months following rural to urban migration[10]. We found a mixed picture, with apparent falls in blood pressure and triglycerides, and increased cholesterol, against a background of increasing weight. Based on these preliminary findings we hypothesised that:

1. Following migration there is a marked decline in physical activity, and this partly accounts for increasing body mass index

2. Following migration there is increased consumption of fresh fruit, vegetables and meat, and these dietary changes partly account for changes in blood pressure and lipids.

3. Increasing body mass index will be related to increasing blood pressure and triglycerides, such that over time any beneficial changes seen in the few months following migration in these factors will start to be reversed.

In this paper we describe changes in the migrants over 12 months, including data on changes in behaviours, such as physical activity and aspects of diet. We examine putative determinants of changes in the biological variables, guided by the above hypotheses, making use of all available data collected at baseline and nine time points following migration.

## Methods

### Overview of the study design

This was a cohort study of rural to urban migrants. It was based in Morogoro rural district, 200 km west of the city of Dar es Salaam, and within Dar es Salaam. The study took advantage of the infrastructure established for a demographic surveillance system, the Adult Morbidity and Mortality Project[11,12]. Through a network of village based key informants, adults who intended to migrate to Dar es Salaam for at least 6 months were identified. Data were collected, by trained observers, at least one week but no more than one month prior to migration, at 2 weeks and 1 month following migration, then at 2, 3, 4, 5, 6, 9 and 12 months after migration. Participants who missed an appointment were visited at their last known residence, on up to at least three occasions per missed appointment if necessary. If no longer resident at that address enquiries were made to other household members and neighbours to try to determine where they had gone, with visits then made to the new location. Ethical approval for the study was obtained from the Tanzanian National Institute of Medical Research.

### Data collection

Height was measured using a Leicester Stadiometer to the nearest half centimetre, and weight measured to the nearest kilogram, without shoes and wearing light clothes, on a seca scale. Body mass index (BMI) was calculated as weight in kilograms divided by height in meters squared. Waist circumference was measured twice using a dress makers tape to the nearest centimetre in the mid-axillary line at the mid point between the lower costal margin and the iliac crest, in gentle expiration with the feet shoulder width apart; the mean of the two measurements was used in analysis. Blood pressure (BP) was measured twice in the right arm with an automated machine (Omron M4), using an alternate size cuff, with the participant seated, after 5 minutes rest and with 5 minutes between measurements. The mean of the two measurements was used in analysis. Fasting blood was taken for plasma lipids, which included total cholesterol, high density lipoprotein (HDL) cholesterol, and triglycerides. These were measured throughout the study in same laboratory at Muhimbili University of Health and Allied Sciences on a Cobas Mira Plus analyser using reagents supplied by the manufacturer. Low density lipoprotein (LDL) cholesterol was estimated using the Friedewald formula[13]. Data collection took place in the morning, and blood samples were kept in a cool box with ice packs and transported and analyzed that day. Transport of samples from the rural area took place by bus, on one of the regular daily services from Morogoro town to Dar es Salaam.

Data on behaviours were collected by interview using questionnaires specifically developed and previously used within Tanzania by the investigators[14,15]. Data were collected on tobacco smoking (including never, ever, and current smoking), and alcohol consumption (type of alcoholic drink, number of drinks per day, week, month or year - which ever was the most frequent), and typical daily physical activity. Individuals were asked to indicate which of five categories best represented their typical daily physical activity, which included for example: spending most of the day sitting (at a desk, at home or outside); spending most of the day on one's feet, carrying, cleaning etc; spending most of one's day digging, hoeing, carrying heavy objects etc. For the analysis the five categories were grouped into three categories, representing typical daily activities as "light", "moderate", and "vigorous". In pilot testing this simple approach to assessing physical activity, which had been used in previous studies, worked much better than a more detailed questionnaire. A twenty item food frequency (never, 1-2, 3-5, 6-7 days per week) questionnaire was administered. This was used to devise scores for the consumption frequency of: fruits and vegetables; carbohydrate; protein; and saturated fat. These scores were used in the multivariable analysis.

Data collected on social and economic circumstances included years of education at primary, secondary and tertiary levels, current main occupation, presence or absence of electricity supply to their residence and source of water supply.

### Statistical Methods

Changes between baseline and 12 months are summarised as means or proportions, differences presented with 95% confidence intervals and statistical significance assessed using the paired T test for continuous variables and McNemar's test for discrete variables. A p value of < 0.05 was considered statistically significant. These analyses of change were performed in the individuals who completed 12 months follow up i.e. had data at baseline and at 12 months.

Mixed linear models with a random intercept[16] were used to explore putative determinants of the change in anthropometry, blood pressure and lipids following migration, using data from all available time points i.e. at baseline and up to 9 time points following migration. Using all the time points, rather than for example baseline and 12 month follow up values, maximises the statistical power of the study[17]. The use of simple linear regression to model only the changes between baseline and follow up was also explored, but fitted the data poorly due to intra individual correlation between the changes following migration. The use of mixed linear models allowed for this intra individual correlation. The models were compared using the Akaike Information Criterion (AIC), and where possible models were fitted to all persons (men and women combined), and if not to men and women separately. Where possible the dietary scores were used, but where these were not significant predictors individual aspects of diet (from the food frequency questionnaire) were examined. A p value of < 0.05 was used to retain variables in the model. The amount of variation accounted for by the final models for inter and intra person variation was assessed by Pseudo R^2 ^Statistics. All analyses were undertaken using the package SAS [18]. Two main models were constructed, one which used all the available data on all available time points, and therefore included individuals who were lost to follow up by 12 months. The second model used all available data on all available time points from only those individuals who completed the 12 months follow up.

## Results

### Migrants recruited and followed up

Table 1 shows the number of migrants by age and sex recruited to the study, plus selected personal and household characteristics. Two hundred and nine migrants, 103 men and 106 women, were recruited to the study and had baseline measurements, prior to migration. Their mean ages (SD) were 28(10) and 30(10) years respectively. Of these individuals, 101 males and 98 females were followed up on arrival in Dar es Salaam. After 12 months follow up had been maintained with 132 (63.2%) of the migrants, 68 (66.0%) men and 64 (60.4%) women ('p value' for differences in follow up by sex = 0.47). There were no significant differences in the baseline characteristics of the 132 with whom contact was maintained and the 77 lost to follow up. For example, mean age was 29.5 vs 27.4 years (95% CIs on the difference -1.6 to 4.5 years, p = 0.15), mean BMI 23.6 vs 23.1 kgm^-2 ^(-0.7 to 1.8 kgm^-2^, p = 0.46), mean systolic blood pressure 124.4 vs 125.9 mmHg (-7.3 to 4.3 mmHg, p = 0.61), and mean cholesterol 4.1 vs 4.2 mmoll^-1 ^(-0.5 to 0.3 mmoll^-1^, p = 0.52) respectively.

**Table 1:** Selected characteristics of the study population at baseline: number (column percent).

	Sex		All
	Male	Female	
**Age group**			
15-29	70 (68.0%)	67 (63.2%)	137 (65.5%)
30-44	21 (20.4%)	23 (21.7%)	44 (21.1%)
45-59	12 (11.7%)	16 (15.1%)	28 (13.4%)
Total	103	106	209
			
**Education**			
< 7 years primary	14 (13.6%)	20 (18.9%)	34 (16.3%)
≥ 7 years primary (no secondary)	76 (73.8%)	77 (72.6%)	153 (73.2%)
Any secondary education	13 (12.6%)	9 (8.5%)	22 (10.5%)
			
**Main economic activity**			
Farming	83 (80.6%)	94 (88.7%)	177 (84.7%)
Other	20 (19.4%)	12 (11.3%)	32 (15.3%)
			
**Religion**			
Muslim	78 (75.7%)	67 (63.2)	145 (69.4%)
Christian	25 (24.3%)	39 (36.8)	64 (30.6%)
			
**Water supply**			
Household tap	9 (8.8%)	13 (12.3%)	22 (10.5%)
Shared	94 (91.2%)	93 (97.7)	187 (89.5%)
			
**Household supplied by electricity**	3 (2.9%)	4 (3.8%)	7 (3.4%)

Column percentages may not add to 100 due to rounding

### Socio economic characteristics before and after migration

The majority of migrants, 69% (95% CIs 63 to 76%), reported their religion as Muslim (Table 1), with the remainder as Christian. At baseline 85% (80 to 90%) described their main occupation as farming. Seventy three percent (67 to 79%) reported at least seven years of primary education but no secondary education, and only 11% (6 to 15) secondary, and none further education. Only 3.3% (0.9 to 5.8%) of participants came from a household supplied by electricity and 11% (6 to 15%) had access to water through a household tap (in their or a neighbour's house), with the rest using a shared water supply (public tap, well or river).

Twelve months following migration, 42% (33 to 50%) still described their main occupation as farming, 19% (12 to 26%) as business, 15% (9 to 21%) as being in education (as a student), and among the 64 women, 20% (10 to 30%) as being a housewife or house girl. Fifty five percent (47 to 64%) of migrants lived in households supplied with electricity and water was available from a household or neighbour's tap for 92% (88 to 97%).

### Changes between baseline and 12 months in behaviours

In both men and women physical activity decreased markedly following migration (Table 2), with the proportions in the highest physical activity category falling by 52.9% (42.7 to 63.2%) in men and 21.9% (11.6 to 32.2%) in women. Weekly alcohol consumption increased from just under a quarter of men to over a third, there was a much smaller, non-significant, increase in women (Table 2). By far the commonest alcoholic drink was bottled beer, with only 2 individuals at 12 months reporting at least weekly consumption of traditional beer, and only 1 individual reporting at least weekly consumption of spirits. No women reported smoking before or after migration while for men there was a non-significant increase in the proportion of regular smokers of 7.4% (-2.2 to 16.9%).

**Table 2:** Physical activity, alcohol consumption and tobacco smoking at baseline and 12 months.

	Baseline	12 months		
Behaviour	%	%	% diff (95% CI)	P value
**Men **(n = 68)				
Alcohol consumed^a^	25.0	36.8	11.8 (-3.8,27.3)	0.138
Current smoking^b^	16.2	23.5	7.4 (-6.1,20.8)	0.282
				
Daily physical activity^c^				
Light	7.4	33.8	26.5 (12.9,40.1)	<0.001
Moderate	13.2	39.7	26.5 (11.6,41.3)	<0.001
Vigorous	79.4	26.5	-52.9 (-69.7,-36.2)	<0.001
				
**Women **(n = 64)				
Alcohol consumed^a^	23.4	29.7	6.3 (-9.1,21.6)	0.423
Current smoking^b^	.	.	.	.
				
Daily physical activity^c^				
Light	28.1	40.6	12.5 (-4.0,29.0)	0.137
Moderate	34.4	43.8	9.4 (-7.5,26.3)	0.277
Vigorous	37.5	15.6	-21.9 (-37.2,-6.6)	0.005

^a^Alcohol consumed at least once a week

^b^Smoking reported within last week

^c^See text for definitions

The diet scores (Table 3) represent the minimum number of "portions" consumed per week. Mean number of "portions" of fruit and vegetables increased in men by 1.8 (0.3 to 3.2) and in women by 3.0 (1.5 to 4.5) per week. The only other notable change in dietary score was saturated fat intake in men, mean increase of 1.8 (0.5 to 3.2) "portions" per week, with a non-significant increase in women despite increased consumption of fried potatoes and red meat.

**Table 3:** Dietary scores and selected aspects of diet at baseline and 12 months.

	start	12 months	Difference	
Food Score	Mean (SD)	Mean (SD)	95% CI	P value
**Men (n = 68)**				
*Diet score*^*a*^				
Fruit & Vegetable Score	9.6 (4.4)	11.3 (5.2)	1.8 (0.3,3.2)	0.017
Protein Score	10.4 (5.6)	10.6 (5.5)	0.3 (-1.5,2.0)	0.77
Carbohydrate Score	22.6 (7.5)	24.3 (8.3)	1.8 (-0.4,3.9)	0.11
Saturated Fat Score	7.5 (4.2)	9.3 (4.7)	1.8 (0.5,3.2)	0.009
				
*Selected food items*^*b*^				
Fried potatoes	1.3 (1.7)	1.8 (1.7)	0.4 (-0.1,1.0)	0.13
Fresh vegetables	1.8 (2.1)	2.7 (2.6)	0.9 (0.1,1.7)	0.022
Fresh fruit	3.4 (2.0)	4.1 (2.2)	0.7 (0.1,1.3)	0.016
Red meat	2.2 (1.7)	3.3 (2.2)	1.1 (0.5,1.7)	0.001
Soft drinks	2.3 (2.2)	3.0 (2.2)	0.7 (0.1,1.3)	0.015
				
**Women (n = 64)**				
*Diet score*^*a*^				
Fruit & Vegetable Score	9.0 (3.6)	12.0 (12.9)	3.0 (1.5,4.5)	<0.001
Protein Score	9.1 (5.6)	9.6 (5.0)	0.5 (-1.3,2.3)	0.57
Carbohydrate Score	22.0 (6.6)	24.4 (8.2)	2.4 (-0.3,5.1)	0.084
Saturated Fat Score	7.2 (4.4)	8.2 (4.5)	1.0 (-0.7,2.6)	0.24
				
*Selected food items*^*b*^				
Fried potatoes	1.0 (1.5)	1.6 (1.8)	0.6 (0.0,1.2)	0.050
Fresh vegetables	1.3 (1.8)	2.7 (2.5)	1.4 (0.6,2.2)	0.001
Fresh fruit	2.8 (2.0)	4.1 (2.3)	1.3 (0.6,2.1)	0.001
Red meat	1.8 (1.6)	2.8 (2.1)	1.0 (0.4,1.6)	0.002
Soft drinks	1.6 (1.8)	2.6 (2.1)	1.0 (0.4,1.6)	0.002

^a^Figures represent the minimum number of "portions" consumed per week.

^b^Figures represent the mean number of days per week on which at least one "portion" of each item was consumed.

### Changes between baseline and 12 months in biological variables

**Figure 1:**
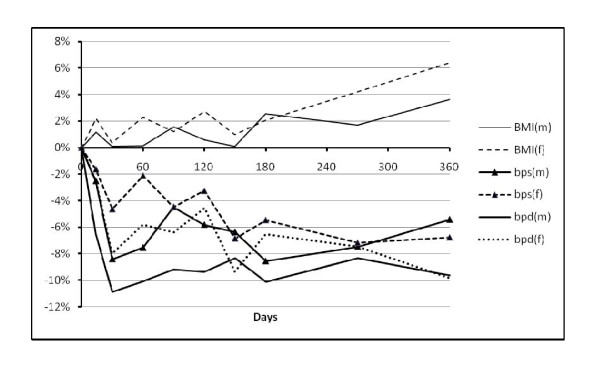
**Percentage change from baseline value over time for body mass index (BMI), systolic blood pressure (sbp) and diastolic blood pressure (dbp) in men (m) and women (f)**.

**Figure 2:**
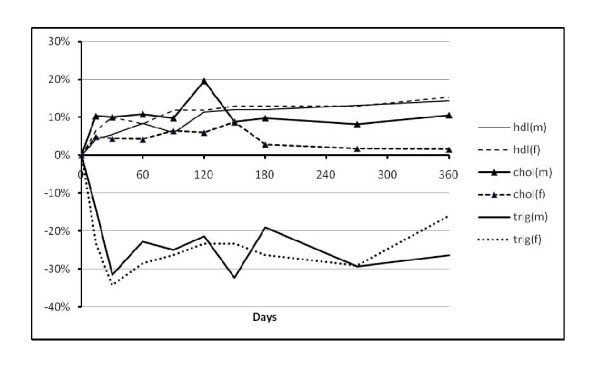
**Percentage change from baseline value over time for cholesterol (chol), HDL cholesterol (HDL) and triglycerides (trig) in men (m) and women (f)**.

The changes in biological variables between baseline and 12 months are shown in Table 4. Figures 1 and 2 show how the variables changed over the course of the 12 months by showing the percentage difference from baseline by days of follow up.

**Table 4:** Anthropometry, blood pressure and lipids at baseline and 12 months by sex following migration.

		Baseline	12 months	difference	
Outcome	N	Mean (SD)	Mean (SD)	95% CI	P value
**Men**					
*Anthropometry*					
Weight (kg)	68	58.8 (10.4)	61.1 (10.3)	2.3 (1.4,3.2)	<0.001
Waist (cm)	68	77.3 (11.2)	79.2 (11.3)	1.9 (1.2,2.5)	<0.001
BMI (kgm-2)	68	21.8 (3.4)	22.7 (3.5)	0.9 (0.5,1.2)	<0.001
					
*Blood Pressure*					
Diastolic BP (mmHg)	68	76.6 (12.1)	68.8 (11.5)	-7.9 (-10.6,-5.1)	<0.001
Systolic BP (mmHg)	68	125.7 (18.7)	120.3 (15.2)	-5.4 (-9.3,-1.6)	0.007
					
*Lipids*					
Cholesterol (mmoll-1)	51	3.74 (1.05)	4.16 (0.99)	0.42 (0.11,0.73)	0.010
HDL Cholesterol (mmoll-1)	51	1.67 (0.31)	1.91 (0.10)	0.24 (0.15,0.33)	<0.001
Cholesterol to HDL ratio	51	2.27 (0.60)	2.18 (0.53)	-0.09 (-0.26,0.08)	0.30
LDL Cholesterol (mmoll-1)	50	1.82 (0.92)	2.06 (0.96)	0.25 (-0.04,0.53)	0.093
Tryglyceride (mmoll-1)	50	1.35 (0.88)	1.04 (0.67)	-0.31 (-0.60,-0.02)	0.034
					
**Women**					
*Anthropometry*					
Weight (kg)	64	61.8 (13.1)	64.1 (12.6)	2.4 (1.3,3.4)	<0.001
Waist (cm)	64	83.3 (11.5)	85.5 (11.4)	2.2 (1.1,3.3)	<0.001
BMI (kgm-2)	64	25.5 (4.8)	26.5 (4.8)	1.0 (0.6,1.4)	<0.001
					
*Blood Pressure*					
Diastolic BP (mmHg)	64	77.7 (13.1)	69.5 (13.5)	-8.2 (-11.7,-4.7)	<0.001
Systolic BP (mmHg)	64	123.0 (21.7)	114.4 (19.2)	-8.6 (-13.7,-3.4)	0.001
					
*Lipids*					
Cholesterol (mmoll-1)	46	4.43 (1.38)	4.67 (1.19)	0.24 (-0.15,0.64)	0.22
HDL Cholesterol (mmoll-1)	45	1.69 (0.40)	1.95 (0.09)	0.25 (0.13,0.37)	<0.001
Cholesterol to HDL ratio	45	2.72 (0.89)	2.41 (0.63)	-0.30 (-0.55,-0.05)	0.019
LDL Cholesterol (mmoll-1)	45	2.46 (1.28)	2.50 (1.15)	0.03 (-0.37,0.44)	0.86
Tryglyceride (mmoll-1)	46	1.40 (0.81)	1.25 (1.12)	-0.15 (-0.47,0.17)	0.35

Includes individuals with data at baseline and at 12 months following migration. Lower numbers with lipids represents failure in some to have a fasting blood sample at baseline and 12 month follow up.

#### Weight, body mass index and waist circumference

Weight, body mass index and waist circumference increased in both men and women (Table 4) e.g. in women weight increased by 2.35 (1.30 to 3.40) kg, BMI by 1.0 (0.55 to 1.44) kgm^-2 ^and waist by 2.18 (1.05 to 3.31) cm. Figure 1 illustrates how average BMI changed over time with an apparent increase in BMI in both men and women from around 4 months.

#### Blood pressure

In both men and women there were falls in systolic and diastolic blood pressure, from a fall in systolic BP in men of -5.4 ( -9.3 to -1.6) mmHg to -8.6 (-13.7 to -3.4) in systolic BP in women. Figure 1 shows that on average the greatest fall in blood pressure occurred within the first month following migration, particularly in men.

As part of exploring the possibility that the fall in blood pressure represented habituation i.e. becoming used to having blood pressure measured, the difference between the first and second blood measurement on each occasion was examined. At baseline the mean difference between the first and second systolic blood pressure measurement in the 132 participants followed up to 12 months was 4.6 (2.5 to 6.8) mmHg, and for diastolic blood pressure it was 1.8 (0.6 to 3.0) mmHg. At 12 months the differences were 0.4 (-1.7 to 2.5) mmHg and 1.5 (-0.1 to 3.1) mmHg respectively. One interpretation of these findings is that at baseline the first blood pressure measurement is strongly influenced by the lack of familiarity with the procedure, particularly for systolic blood pressure. Given this, the change in blood pressure between baseline and 12 months was also examined using the second reading only (rather than the mean of the first and the second).

When only the second blood pressure reading is used the difference between baseline and 12 months in men was -8.1 (-5.2 to -11.1) mmHg for diastolic blood pressure, and -3.9 (0.6 to -8.3) mmHg for systolic blood pressure; with equivalent values in women of -7.6 (-3.7 to -11.4) mmHg, and -5.9 (-0.3 to -11.4). Thus, using only the second reading the findings were qualitatively similar, but with a smaller, and in men non-significant, fall in systolic blood pressure. In addition, the second reading at baseline was compared with the first reading at 12 months, and the findings were little different.

The change in blood pressure was also examined after excluding those who reported a diagnosis of hypertension and taking blood pressure lowering medication. There were two such individuals at baseline (one male, one female), and 9 individuals (2 males, 7 females) at the 12 month follow up. Excluding these individuals from the analysis made little difference to the fall in blood pressure between baseline and 12 months, being for example -5.2 (-9.3 to -1.3) mmHg for systolic blood pressure in men, and -6.6 (-11.5 to -1.7) mmHg for systolic blood pressure in women.

Finally, the difference in blood pressure between baseline and 12 month follow up was examined by higher (greater than or equal to 140 systolic and/or 90 mmHg) or lower blood pressure at baseline (Table 5). Those with higher blood pressure at baseline had the greatest falls in blood pressure. In those with baseline blood pressure <140/90 there were significant falls in diastolic blood pressure only.

**Table 5:** Changes in blood pressure according to baseline values below and above 140/90.

	N	Fall in blood pressure (95% CIs) (mmHg)	P value	12 month value as % of baseline
**Men**				
*Systolic < 140 and diastolic < 90*				
Diastolic BP	54	-5.6(-8.4, -2.8)	<0.001	93
Systolic BP	54	-0.1 (-3.5, 3.2)	0.90	100
*Systolic ≥ 140 or diastolic ≥ 90*				
Diastolic BP	14	-16.8 (-22.9, -10.3)	<0.001	81
Systolic BP	14	-25.9 (-32.5, -19.3)	<0.001	83
				
**Women**				
*Systolic < 140 and diastolic < 90*				
Diastolic BP	51	-6.7 (-10.5, -2.9)	0.001	91
Systolic BP	51	-4.1 (-9.1, 0.94)	0.11	96
*Systolic ≥ 140 or diastolic ≥ 90*				
Diastolic BP	13	-14.0 (-23.3, -4.7)	0.006	86
Systolic BP	13	-26.0 (-39.0, -13.1)	0.001	84

#### Lipids

Due to the lack of a fasting sample at either baseline or 12 months, lipid results were missing on 17 men and 18 women (Table 4). In both men and women with lipid values, HDL cholesterol increased between baseline and 12 months, by 0.24 (0.15 to 0.33)mmoll^-1 ^and 0.25 (0.13 to 0.37) mmoll^-1^respectively. In women there was little change in total cholesterol and cholesterol to HDL ratio fell (-0.3, -0.55 to -0.05), where as in men total cholesterol increased (0.42, 0.11 to 0.73 mmoll^-1^) and there was little change in HDL to cholesterol ratio. Serum triglycerides fell significantly in men at 12 months (-0.31, -0.6 to -0.02 mmoll^-1^) but not in women. However, as Figure 2 shows triglyceride levels in women initially fell as much, if not more, than those in men. At six months, for example, the mean change in serum triglycerides in women was -0.45 (-0.20 to -0.71) mmoll^-1^, but reduced to a non-significant difference towards the end of the 12 month follow up.

### Predictors of change in biological variables

Table 6 shows the results of the mixed linear models, using all the available data, including data on individuals who did not complete 12 months follow up, aiming to account for inter and intra individual variation in BMI, blood pressure and lipids. Age and aspects of diet contributed to the model for BMI, and age and BMI contributed to the models for systolic and diastolic blood pressure, total cholesterol, triglycerides, and LDL cholesterol in women. Various aspects of diet were additional contributors to the models. For example, consumption of traditional porridge was negatively associated with systolic and diastolic blood pressure, and BMI in men. Fruit and vegetable score was positively associated with both triglycerides and HDL cholesterol. The models were reasonably good at accounting for the inter-individual variance in systolic and diastolic blood pressure, triglycerides and total cholesterol. However, the models largely failed to account for intra-individual variance (i.e. variance over time). For example, the models accounted for between 25% and 40% of the inter-individual differences in total cholesterol, triglycerides, and systolic and diastolic blood pressure, but only at best accounted for 2.1% of intra-individual differences (triglycerides in women). These analyses were repeated, limiting them to data from the 132 individuals who were followed up to 12 months. Qualitatively the findings were virtually identical, with some minor increases in the width of confidence intervals on the regression coefficients and increased size of p values (data not shown, but available on request).

**Table 6:** Predictors of BMI, blood pressure, and lipids, and variance accounted for (R^2^) for inter-person and intra-person differences.

Outcome	Male/Female	Variables in Model (p < 0.05)	Standardised Coefficient with		Pseudo R^2^	
			95% CI	P value	Inter-person	Intra-person
**BMI**	Men	Age (unit = 10)	0.27(0.08,0.46)	0.006	6.4%	2.2%
		Porridge	-0.03(-0.05,-0.01)	0.013		
		Peanuts	-0.03(-0.05,-0.01)	0.011		
	Women	Age (unit = 10)	0.28(0.10,0.46)	0.003	7.9%	0.9%
		Boiled potatoes	0.02(0.00,0.03)	0.019		
**Systolic BP**	Men & women	Age (unit = 10)	0.37(0.27,0.46)	<0.001	35.2%	0.7%
		Female sex	-0.16(-0.26,-0.07)	0.001		
		BMI	0.16(0.07,0.25)	0.001		
		Margarine on bread	-0.06(-0.10,-0.02)	0.006		
		Porridge	-0.04(-0.08,-0.01)	0.024		
**Diastolic BP**	Men & women	Age (unit = 10)	0.37(0.28,0.46)	<0.001	39.5%	1.3%
		BMI	0.16(0.07,0.24)	<0.001		
		Margarine on bread	-0.08(-0.12,-0.04)	<0.001		
		Fried potatoes	-0.05(-0.09,-0.01)	0.014		
		Porridge	-0.04(-0.08,0.00)	0.046		
**Total cholesterol**	Men & women	Age (unit = 10)	0.20(0.10,0.31)	<0.001	25.3%	0.1%
		Female sex	0.14(0.03,0.24)	0.009		
		BMI	0.16(0.07,0.26)	0.001		
		Biscuits	-0.04(-0.09,0.00)	0.045		
**HDL cholesterol**	Men & women	Age (unit = 10)	0.14(0.05,0.23)	0.004	6.8%	0.4%
		Fruit and veg score	0.07(0.01,0.12)	0.012		
**LDL cholesterol**	Men	Age (unit = 10)	0.17(0.03,0.31)	0.015	8.8%	1.5%
		Red meat	0.07(0.01,0.14)	0.034		
		Biscuits	-0.09(-0.16,-0.02)	0.011		
	Women	Age (unit = 10)	0.05(0.02,0.07)	0.001	21.0%	0.8%
		BMI	0.89(0.17,1.60)	0.015		
		Processed meat	0.06(0.01,0.12)	0.023		
**Triglycerides**	Men	Age (unit = 10)	0.21(0.09,0.33)	0.001	25.8%	1.7%
		BMI	0.13(0.02,0.24)	0.026		
		Fried potatoes	-0.10(-0.18,-0.02)	0.011		
		Sweet snacks	0.08(0.00,0.15)	0.040		
	Women	Age (unit = 10)	0.20(0.08,0.32)	0.001	34.9%	2.1%
		BMI	0.21(0.10,0.32)	0.000		
		Fruit and veg score	0.09(0.01,0.16)	0.022		
		Saturated fat score	-0.08(-0.16,0.00)	0.050		

Results from multivariable analysis with mixed linear models in which all available data points for each individual, at baseline and following migration, were used. If possible, models were fitted for men and women combined, where not possible they were fitted separately.

Potential predictors considered for BMI were age, physical activity, dietary score/dietary items.

Potential predictors considered for all other outcomes were age, sex (male and female combined models) BMI, physical activity, dietary score/dietary items, alcohol consumption.

Dietary scores were entered first but where none of the scores were significant predictors individual dietary items were then evaluated.

## Discussion

In this study we set out to investigate changes and their determinants in cardiovascular and diabetes risk factors in the year following rural to urban migration in Tanzania. Following migration we found some changes that would be predicted by published cross sectional data, and others that seem contrary to them. Thus, as expected physical activity decreased markedly, and there was evidence of saturated fat consumption increasing. However, there was also evidence that consumption of fresh fruit and vegetables also increased. As expected, over 12 months following migration weight and waist circumference increased, and total cholesterol significantly increased in men, but did not in women. Contrary to expectation, HDL cholesterol increased in both men and women, and in women the ratio of total to HDL cholesterol fell. In men, but not women, there was a significant fall in triglycerides over 12 months. Blood pressure fell in both men and women.

Before attempting to appraise the importance of the findings from this study, it is crucial to acknowledge its strengths and limitations. Strengths of this study include the fact that participants were recruited, with measures made, prior to migration, and that a reasonable proportion (63%), particularly for the challenging nature of the urban environment, were followed to the end of the study. There were not systematic differences at baseline between those lost to follow up and those not. The inclusion of lipids, the first migration study as far as we are aware to have done so in sub Saharan Africa, is also a strength. The lipids at baseline were transported on the same day from the rural area to the laboratory in the urban area, a two to three hour bus journey, and realistically half a day from taking the sample to separating and analysing the sample in the laboratory. However, cholesterol and triglycerides are highly stable analytes, even after, for example, with the sample being left for at least 24 hours at 35 degrees centigrade[19]. Limitations of the study include its relatively small sample size and the relatively crude assessment of some determinants, particularly diet and physical activity. The instruments we used to collect these data had worked well under field conditions in previous studies in Tanzania[14,15], and were chosen after piloting more complex diet and physical activity questionnaires, which worked poorly. However, more precise and objective measures on larger numbers would clearly provide greater confidence on both the size and variances of changes. Our inability in multivariable analysis to account for any of the intra-individual changes in biological variables following migration may in large part be a result of crude measures of physical activity and diet.

Despite the obvious limitations we believe that this study provides important and novel insights. Blood pressure did not rise following migration. The fall in blood pressure was unexpected. It was not explained by treatment of hypertension following migration, occurred in both those with higher and lower blood pressure at baseline, and was apparent whether the mean of the blood pressure readings taken on each occasion was used or only the second reading. Nonetheless, it seems likely that much of the fall we found in blood pressure is the result of habituation, with increasing familiarity and less anxiety on repeated measures. A previous study in residents (not migrants) of Dar es Salaam[20] evaluated the impact of repeated measurements on blood pressure, and found that over four visits over the course to 6 to 7 weeks, average blood pressure fell markedly, with the greatest falls in those with the highest initial blood pressure. The falls we found in blood pressure (Table 5) were similar. In addition, the measurements in the rural area were made in participants' homes, while in the urban area they were made at clinics. A small study from rural Kenya in the early 1980s found that blood pressure tended to be higher (on average 2 to 3 mmHg) when measured at home compared to the clinic[21]. The reasons for this difference were not clear, and we do not know if they apply to the population and settings of this study. However, in conclusion habituation and perhaps home based versus clinic based measurements are likely to account for much if not all of the fall in blood pressure that we found. Nonetheless it is noteworthy that blood pressure did not rise following migration, as it did in the Kenyan Luo study[22], where sustained increases in blood pressure within one month of rural to urban migration were observed.

As described above, we found mixed changes in lipid levels. A plausible explanation for these changes are changes in diet[23], with our data suggesting a more varied diet in both men and women, particularly with increased fresh fruit and vegetables in men and women, and higher saturated fat intake in men. While our dietary assessment was relatively crude, the broad changes we describe are consistent with detailed cross sectional data from South Africa comparing rural, peri urban and urban communities, that found that the most varied diet, and that richest in fresh fruit and vegetables was in the urban area[24]. Increased fresh fruit and vegetables, assuming similar sodium intake, would also be expected to have beneficial effects on blood pressure[23].

## Conclusions

In contrast to the perception of urban living in low and middle income countries being universally detrimental for cardiovascular risk, the findings from this study suggest that a more nuanced understanding may be appropriate, at least in some settings. Urban compared to rural living may offer some advantages, including access to a more varied diet, but this is against a background of lower levels of physical activity and rising overweight and obesity. A better understanding of how risk changes on migration is central to considering measures to prevent the rising levels of diabetes and cardiovascular disease in low and middle income countries.

## Competing interests

The authors declare that they have no competing interests.

## Author contributions

NU was involved in the design and management the study throughout, and led the writing of the paper; PJ assisted with data management, carried out all the statistical analyses and drafted relevant sections of the paper; DM was the project manager in Tanzania and coordinated all aspects of the data collection and data entry; HM was the principal investigator in Morogoro, and contributed to the detailed study protocol and greatly facilitated its local implementation; PN and MN both contributed to the development of the detailed study protocol and oversaw its implementation in Morogoro; BT contributed to the development of the detailed study protocol and in particular oversaw the biochemical assays at Muhimbili National Hospital; RM provided detailed input into the design, execution and interpretation of the statistical analyses. All authors approved the submitted version of the paper.

## Pre-publication history

The pre-publication history for this paper can be accessed here:

http://www.biomedcentral.com/1471-2458/10/272/prepub
